# Disparate effects of antibiotic-induced microbiome change and enhanced fitness in *Daphnia magna*

**DOI:** 10.1371/journal.pone.0214833

**Published:** 2020-01-03

**Authors:** Asa Motiei, Björn Brindefalk, Martin Ogonowski, Rehab El-Shehawy, Paulina Pastuszek, Karin Ek, Birgitta Liewenborg, Klas Udekwu, Elena Gorokhova

**Affiliations:** 1 Department of Environmental Science & Analytical Chemistry (ACES), Stockholm University, Stockholm, Sweden; 2 Department of Molecular Biosciences, The Wenner-Gren Institute, Stockholm University, Stockholm, Sweden; 3 Aquabiota Water Research AB, Stockholm, Sweden; 4 Swedish University of Agricultural Sciences, Department of Aquatic Resources, Institute of Freshwater Research, Drottningholm, Sweden; Universidade Federal do Rio de Janeiro, BRAZIL

## Abstract

It is a common view that an organism’s microbiota has a profound influence on host fitness; however, supporting evidence is lacking in many organisms. We manipulated the gut microbiome of *Daphnia magna* by chronic exposure to different concentrations of the antibiotic Ciprofloxacin (0.01–1 mg L^-1^), and evaluated whether this affected the animals fitness and antioxidant capacity. In line with our expectations, antibiotic exposure altered the microbiome in a concentration-dependent manner. However, contrary to these expectations, the reduced diversity of gut bacteria was not associated with any fitness detriment. Moreover, the growth-related parameters correlated negatively with microbial diversity; and, in the daphnids exposed to the lowest Ciprofloxacin concentrations, the antioxidant capacity, growth, and fecundity were even higher than in control animals. These findings suggest that Ciprofloxacin exerts direct stimulatory effects on growth and reproduction in the host, while microbiome- mediated effects are of lesser importance. Thus, although microbiome profiling of *Daphnia* may be a sensitive tool to identify early effects of antibiotic exposure, disentangling direct and microbiome-mediated effects on the host fitness is not straightforward.

## Introduction

In multicellular organisms, the microbiome contributes to critical aspects of host development and physiology [1]. In studies on microbiome-host interactions, there is growing recognition that environmental stresses imposed upon the microbiome may drive physiological responses, life-histories, and adaptation capacity of their hosts [2–4] at various environmental settings. Consequently, coping with environmental stressors would involve both the host and its microbiome responses.

The gut microbiota participates directly in food digestion and nutrient assimilation, which affects the host’s energy acquisition and growth [5]. In addition to this, the host immune system is influenced by the gut microbes via a number of different mechanisms, e.g., competition with pathogens as well as suppression and modification of virulence factors via metabolite production [6]. Symbiotic bacteria are also capable of enhancing the host innate immune system by, for example, up-regulation of mucosal immunity, induction of antimicrobial peptides and antibodies [7, 8]. Considering the biological effects triggered by the host-microbiome interactions, a disruption of mutualistic bacterial communities may result in increased susceptibility to pathogens and infections, while simultaneously affecting the growth and development of the host via compromised nutrition. In various gnotobiotic animal models, poor survival, growth and fecundity are commonly observed, reflecting a physiological impairment due to some dysbiotic state of microflora [3, 9].

If growth penalties are to be expected in animals with perturbed microbiota, then it should be possible to manipulate animal fitness by targeting its resident bacteria with antibacterial substances. In line with this, retarded development has been observed in the copepod *Nitocra spinipes* upon antibiotic exposure and linked to structural changes in its microbiota [10]. It was suggested that aberrant digestion was behind these changes as has also been observed in *Daphnia magna* following a short-term antibiotics exposure [9, 11]. Moreover, long-term exposure to the antibiotic oxytetracycline altered microbiota composition in *Daphnia* in a dose-dependent manner, concurrent with changes in host body size [12]. While perturbed microbiota can manifest itself directly as decreased nutrient uptake, another outcome can be decreased antioxidant production by the host, with concomitant impairment of immunity, metabolism, and growth [13]. However, short antibiotic exposure and changes in oxidative status may not necessarily result in any significant growth penalties in the long run. The outcome of any chronic exposure to antibiotics would largely depend on the resilience of the bacterial communities, and their capacity to recover and re-establish functional interactions with the host [14–17].

To study the relationships between microbiome composition and host performance, a common set of model species and methods to manipulate their microbiomes is needed. In ecology, evolution, and ecotoxicology, *Daphnia* species are used routinely as model organisms because of their well-known physiology, rapid reproduction, and sensitivity to environmental factors [18,19]. The microbiome of the laboratory-reared *Daphnia magna* has been recently described in several studies using different approaches, from cloning to shotgun sequencing [20,21]. Regardless of the sequencing platform, origin of specimens, and culture conditions, the core microbiome appears relatively stable, particularly at higher-rank taxonomy, mainly comprised of Betaproteobacteria, Gammaproteobacteria and facultative anaerobic Bacteroidetes species. At the genus level, *Limnohabitans* has been reported as one of the most stable and dominant gut microbiota members in *Daphnia*; moreover, variation in its abundance has been positively related to the animal fecundity [22]. Although some studies have addressed the dependence of *Daphnia* on its microbiota [9], including short-term effects on fitness following exposure to antibiotics in *Daphnia magna* [23,24], the relationships between microflora perturbation and host fitness are still unclear, as is the involvement and modulating role of antioxidants in the host responses.

In this study, the relationship between antibiotic-mediated gut microbiome changes and host fitness were addressed experimentally using a model cladoceran *Daphnia magna*. We monitored changes in the gut microbiome, host longevity, growth, and reproduction, as well as antioxidant capacity in the animals following Ciprofloxacin exposure. We hypothesized that the diversity of the gut microflora and relative abundance of the core taxa would decrease with increasing Ciprofloxacin concentration. Furthermore, we expected longer exposure time and higher antibiotic concentrations to have negative effects on somatic growth, reproductive output, and antioxidant capacity. These reductions would be due to reduced bacterial diversity, and to some extent, changes in the community composition. These hypotheses were tested by combining (1) long-term (21 d) exposure experiments with life-table analysis, (2) microbiome profiling using the next generation sequencing of 16S rRNA gene and taxonomic assignment, and (3) measurements of daphnid total antioxidant capacity, growth, and fecundity.

## Material and methods

### Test species and culture conditions

The cladoceran *Daphnia magna*, originating from a single clone (Environmental pollution test strain *Clone 5*, Federal Environment Agency, Berlin, Germany), was used in this experiment. The animals were cultured in groups of 20 individuals in 3-L beakers with M7 medium as recommended by OECD guidelines 211[25], and fed a mixture of the green algae *Pseudokirchneriella subcapitata* and *Scenedesmus subspicatus* three times a week; the algae were grown axenically.

### Ciprofloxacin stock solutions

We used Ciprofloxacin hydrochloride (CAS: 86393-32-0; Sigma), a broad spectrum fluoroquinolone, active against both Gram-positive, G+, and Gram-negative, G-, bacteria. Its mode of action is the inhibition of the gyrase and / or topoisomerase enzyme of microbes which determines the supercoiling state of DNA, and critical to bacterial replication, repair, transcription and recombination [26]. Selection of this drug was due to its rapid absorption and long half-life in the test system. The exposure concentrations were chosen based on (*i*) reported concentrations in effluents [27] and waste waters [28] corresponding to the lowest test concentration, (*ii)* absence of acute toxicity for *D*. *magna* within the range of concentrations tested [29], and (*iii*) minimum inhibitory concentrations for a range of bacteria [30], representing the entire range of the test concentrations. A stock solution of Ciprofloxacin (1 mg L^-1^) was prepared in M7 medium, stored at -20°C, and used during the experiment.

### Experimental design

We employed three Ciprofloxacin concentrations (0.01, 0.1 and 1 mg L^-1^) and a control treatment (M7 medium). For each treatment, 25 neonates (< 24 h) of *D*. *magna* were placed individually in 40 mL of M7 medium, with or without Ciprofloxacin; the medium was changed every second day. The test design followed the guidelines for the reproduction test with *Daphnia* (OECD standard 211) [25]. The animals were fed daily with a suspension of green algae *Pseudokirchneriella subcapitata* (0.2 mg C d^-1^; axenic culture) and incubated at 22°C with 16^L^: 8^D^ photoperiod. Under these conditions, the animals matured and started to reproduce 8–9 d after the start of the experiment. All jars were inspected daily and mortality was recorded. Upon release of neonates, the brood size was recorded, and the offspring were discarded. In conjunction with brood release, four randomly selected individuals from each treatment were sampled for microbiome analysis. Their images were acquired by scanning live animals on a glass surface in a drop of sterile water (CanoScan 8800F 13.0), and their body length (BL, mm) was measured using ImageJ software [31]. For each individual, the gut was dissected using a sterile needle and a pair of forceps, washed with nuclease-free water, transferred individually to Eppendorf tubes and stored at −80°C until DNA extraction. The degutted body was transferred to an Eppendorf tube and stored at −80°C; these samples were used for measurements of total antioxidant capacity and individual protein content. In this manner, we collected and analyzed females after their 1^st^, 2^nd^, 3^rd^, and 4^th^ clutch, with the last individuals sacrificed on day 21, when the experiment was terminated.

### DNA extraction

DNA was extracted from the gut samples using 10% Chelex [32] and purified with AMPure XP beads (Beckman Coulter, Brea, CA, USA) following the manufacturer’s instructions. After the purification, the DNA concentrations were measured using Quant-iT PicoGreen dsDNA Assay kit (ThermoFisher, USA) as specified in the method description [33]. Absorbance was measured at 530 nm, using a Tecan Ultra 384 SpectroFluorometer (PerkinElmer, USA).

### 16S rRNA gene amplification and sequencing library preparation

Bacterial diversity of the samples was analyzed by sequencing amplicons generated from the V3-V4 region of the 16S rRNA gene using the MiSeq Illumina platform. Two-stage PCR amplification was performed using forward primer 341F (CCTACGGGNGGCWGCAG) and reverse primer 805R (GGACTACHVGGGTWTCTAAT). The first PCR was carried out in 25-μl PCR reactions and comprised 0.02 U μl^-1^ Phusion polymerase (ThermoFisher, USA), 0.2 mM dNTP, 1 mM MgCl_2_, 1 × Phusion reaction buffer, 0.5 μM of each primer as well as 5 ng of DNA template. The amplification protocol consisted of an initial denaturation at 98°C for 30 seconds followed by 35 cycles of 10 sec at 98°C, 30 sec at 55°C and 72°C, and, a final extension step (72°C for 10 min). PCR products were purified using Agencourt AMPure XP beads (Beckman Coulter, Brea, CA, USA). Following this, amplicon PCR was performed on 5 μl of equimolar amounts of PCR product using Nextera XT primers (Index 1 [N7XX] and Index 2 [S5xx]), targeting the same region of the 16S rRNA genes (8 cycles of 30 sec at 95°C, 30 sec at 55°C and 35 sec at 72°C). The products were purified with Amplicons AMPure XP Beads (Beckman Coulter) according to the manufacturer protocol and concentrations were estimated using Quant-iT PicoGreen dsDNA Assay kit (ThermoFisher, USA). Individually barcoded samples were mixed in equimolar amounts, and DNA sequencing adaptor indexes ligated using the TruSeq DNA PCR-free LT Library Preparation Kit (Illumina). Quality control was performed on an Agilent 2100 BioAnalyser using high sensitivity DNA chip. PhiX DNA (10%) was added to the denatured pools, and sequencing was performed on an Illumina MiSeq using the MiSeq V3 reagent kit (600-cycles) on the Illumina MiSeq platform. De-multiplexing and removal of indexes and primers were done with the Illumina software v. 2.6.2.1 on the instrument according to the standard Illumina protocol.

### Sequence data processing

Following initial upstream de-multiplexing and index removal, sequences were analysed using the *DADA2* v. 1.6 module [34] as implemented in the R statistical software v. 3.4.2 [35]. The pipeline consisted of quality-filtering, trimming of bad quality (< Q30) stretches, error estimation and de-replication of reads, merging of forward and reverse reads and finally, removal of chimeric sequences. All remaining sequences were assigned taxonomy on the genus level using the Silva Ribosomal RNA database version v.128. Subsequent statistical analyses and visualization were done with the *Phyloseq* R-module v.1.22.3 [36] unless otherwise stated. The data has been deposited with the following accession-number PRJNA560134: DaphniaABeffects at NCBI.

### Analysis of oxygen radical absorbance capacity and protein content

As a proxy for antioxidant capacity, we assayed oxygen radical absorbance capacity (ORAC) according to [37] with minor modifications; the measured values were normalized to the individual protein content. This biomarker represents the water-soluble fraction of antioxidants and has been applied for analysis of antioxidant production in daphnids [38]. Samples for ORAC and protein measurements were homogenized in 100 μL of PPB buffer (75 mM, pH 7.4). Fluorescein was applied as a fluorescent probe (106 nM) and 2, 2- azobis (2-amidinopropane) dihydrochloride (AAPH) (152.66 mM) as a source of peroxyl radicals. Trolox (218 μM, Sigma–Aldrich) was used as the standard. The assay was conducted in 96-well microplates while 20 μL of homogenate sample was added to each well and mixed with 30 μL of AAPH and 150 μL of fluorescein. Fluorescence was measured at 485nm/520nm (excitation/emission wavelength).

Protein content of the supernatant was determined by the bicinchoninic acid method using a Pierce BCA Protein Assay kit 23227 (ThermoFisher, USA) according to the microplate procedure with some modifications. In each well, 25 μl of blank, standard or samples was added to 200 μl of working solution. Absorbance was measured at 540 nm using a FluoStar Optima plate reader (BMG Lab Technologies, Germany). Antioxidant capacity was expressed as mg Trolox eq. mg protein^−1^.

## Data analysis and statistics

### Life-history traits

Survival probability was calculated using Kaplan-Meier analysis, which estimates the probability of an event (i.e., death) occurring in a given period [39]. The logrank test was used to evaluate differences in the survivorship among the treatments using package *survival* in R [40].

The empirical von Bertalanffy growth model was applied to determine growth parameters using length-at-age data fitted to the equation:

$$BL={BL}_{max}\times (1-{exp}^{(-K\times t})$$ Eq 1

where *BL* is the total length at time *t* (days); *BL*_*max*_ is the length reached at an infinite time, defined as the maximum potential length attained under the prevailing conditions; and *K* is the individual growth rate. Statistical differences in *BL*_*max*_ and *K* between each treatment and control were determined by non-overlapping 95% confidence intervals.

To analyze the effects of exposure time and Ciprofloxacin concentration on the daphnid fecundity, we used generalized linear models (GLM) with Poisson distribution and identity link function. Residuals were checked visually, and nonsignificant interaction terms were dropped from the analysis. A post hoc Tukey HSD test was used to compare the brood size among the treatments for each clutch.

The daphnid population growth rate (*r*) was estimated according to Euler-Lotka’s equation using (R Core Team, 2018) (S1 File)

$$\sum_{x=\alpha }^{\beta }l(x)\;m(x)e^{-rx}=1$$ Eq 2

where *l(x)* is the fraction of individuals surviving to age *x* and *m(x)* is the birth rate per capita for the mothers of age *x*. Bootstrapping (999 permutations) was used to estimate 95% confidence limits of the *r* values in each treatment, and statistical differences in *r* between each treatment and control were determined by non-overlapping 95% confidence intervals.

### Microbial communities

To assess the alpha diversity of the bacterial communities, we calculated commonly used indices (Shannon-Weiner, ACE, Chao1 and Fisher´s alpha) that consider both richness and evenness to describe the diversity of a community. The indices were calculated using individual data rarefied to equal sequencing depth at treatment level. Rarefaction curves was plotted using functions supplied by the *vegan* R-libraries. Zhang Huang’s index was calculated using OTU abundance data and *entropart* package. This index is used to validate the coverage-based community richness instead of size-based rarefaction to avoid biased comparison of communities with many rare species [41].

Effects of Ciprofloxacin concentration and time on the diversity indices were evaluated using generalized linear models (GLM) with normal error structure and log-link. Quantile plots were used to evaluate the distribution of the residuals and deviance was used to access goodness of the model. Interaction (*time* × *concentration*) was first included in every model but omitted if found not significant.

The Principal coordinates analysis (PCoA) with Bray-Curtis dissimilarity index was used to visualize differences in community composition among the treatments [42]. Differences in the community structure at the family level were tested by permutational multivariate analysis of variance (PERMANOVA) Bray-Curtis dissimilarity was used as variance stabilizing transformation. Multivariate homogeneity of treatment dispersion was assessed using the *betadisper* function in the *vegan* package [43].

A heatmap of core microbiome, a set of bacteria consistently present in the host, was generated using R-package *Microbiome* version 1.1.2; the prevalence was set at 20% and detection threshold at 0.01%. Moreover, to examine and visualize the core microbiome members shared among the microbial communities and unique OTUs among the treatments, a Venn diagram was generated using package *Venndiagram* and the rarefied OTUs after applying low count filter of 4 reads with prevalence of 20% in each sample. Shared taxa present in all four groups (100% core threshold) were defined as the core microbiome.

### Linking microbiome to host fitness

The R-package *edgeR* [44] was used to identify differentially abundant bacterial taxa (false discovery rate-corrected *p*-values, α = 0.05, FDR = 1%) that were associated with high or low growth rate (somatic and reproductive) of the daphnids. As a measure for somatic and reproductive growth, we used body length (BL) and fecundity, respectively. For each trait, we created two classes, *high* (above the group mean, coded as 1) and *low* (below the group mean, coded as 0) using zeta scores for individual BL and fecundity measurements. Zeta scores (zero mean, unit variance normalization) were calculated based on clutch-specific mean values (all treatments included) and corresponding standard deviations to account for the changes in BL and fecundity with the daphnid age.

## Results

### Survival and individual growth

**Fig 1:**
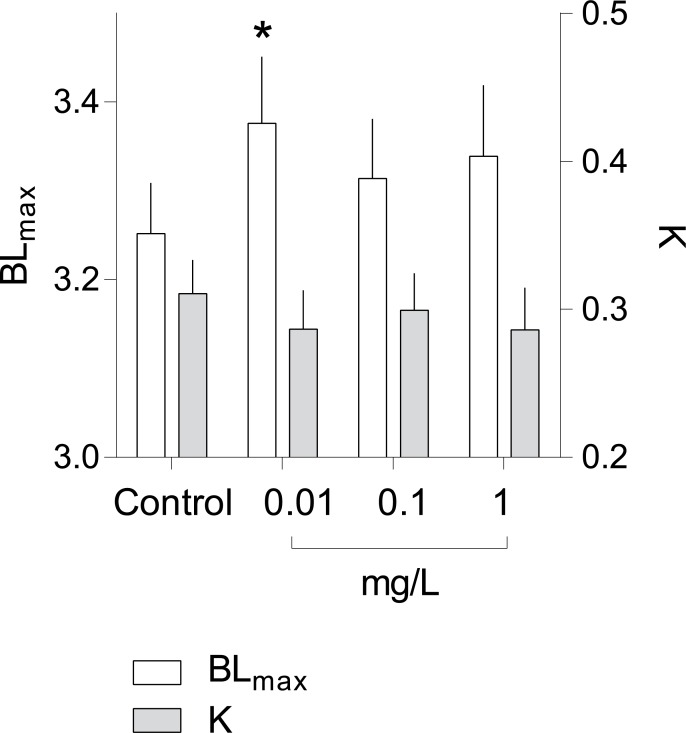
Individual growth curves analyzed by empirical von Bertalanffy model. Estimated *BL*_*max*_ and *K* values (Eq 1) and corresponding 95%-confidence limits for *Daphnia magna* grown in 0.01, 0.1 and 1 mg L^-1^ Ciprofloxacin and the control.

The survival rate was moderate to high (84% to 92%), not differing significantly among the treatments (log rank test, *p* > 0.8; all treatments included), although the antibiotic-exposed animals had slightly higher survival compared to the controls (S1 Fig). According to the growth curve analysis, the animals exposed to the lowest Ciprofloxacin concentration (0.01 mgL^-1^) had a significantly greater maximal body length (BL _max_) compared to the controls, whereas the individual growth rate (K) was similar across the treatments (Fig 1, Table 1).

**Table 1:** Body growth parameters estimated by von Bertalanffy model.

Best-fit values	1 mg/L	0.1 mg/L	0.01 mg/L	Control
*BL*_*ma*x_	3.339	3.314	3.376	3.252
*K*	0.286	0.299	0.287	0.311
**SE estimates**				
*BL*_*ma*x_	0.039	0.033	0.037	0.028
*K*	0.014	0.012	0.013	0.011
**95% Confidence Intervals**				
*BL*_*ma*x_	3.259 to 3.419	3.247 to 3.381	3.302 to 3.451	3.195 to 3.309
*K*	0.2575 to 0.3146	0.2743 to 0.3244	0.2604 to 0.3130	0.2880 to 0.3333
**Goodness of Fit**				
R^2^	0.989	0.992	0.991	0.993
df	30	30	30	30
Sum of Squares	0.509	0.369	0.440	0.292
Sy.x	0.130	0.111	0.121	0.099

The model parameters (*BL*_*ma*x_ and *K*) were estimated according to Eq 1 using BL measurements of the animals exposed to Ciprofloxacin (0.01, 0.1 and 1 mg L^-1^) and those in the control.

### Reproduction

The average brood size was significantly higher in all Ciprofloxacin treatments compared to the control (GLM, t_263, 267_ = 12.97,*p* < 0.001; S2 Fig), with the increase varying from 36% in the 0.01 mg L^-1^treatment (t_263, 267_ = 4.347; *p* < 0.001) to 42% in the 0.1 mg L^-1^ treatment (t_263, 267_ = 4.05; *p* < 0.001). Also, there was a significant negative effect of *time* (t_263, 267_ = -2.74; *p* < 0.05), which was mainly related to the low values in the last brood (Tukey HSD, z _(4–1)_:-3.084, *p*_*(*4–1)_ < 0.01; z _(4–2)_: -5.97, *p*_(4–2)_ < 0.01; z _(4–3):_ -3.34, *p*_(4–3)_ <0.005; numbers in brackets refer to the clutch number).

### Population growth rate

The population growth rate varied from 0.26 to 0.30 among the treatments and was higher in the exposed daphnids relative to the control by 17%, 19% and 15% in the animals exposed to 0.01, 0.1 and 1 mgL^-1^, respectively. The differences from the control were significant for all treatments (S1 Table).

### Characterization of the gut microbiota in *Daphnia*

A total of 1314 OTUs were obtained after filtering out reads with low-quality and removal of chimera and contaminant sequences. Rarefaction curves plateaued with the current sampling effort and Zhang Huang’s index was high (99.8 ± 0.001%, mean ± SD) across the treatments indicating that the bacterial communities were adequately sampled (S3 Fig). The gut microbiome of our test animals was dominated by Proteobacteria, which contributed on average 74% (ranging from 25% to 95% in individual specimens). When all treatments were considered, Actinobacteria (15%), Bacteroidetes (7%), Firmicutes (1%) and Verrucomicrobia (1%) were also common. In the non-exposed animals, the contributions were different, with Proteobacteria, Bacteroidetes and Verrucomicrobia being the most common (S4 Fig). Together, these five phyla formed the core microbiome of the gut (S5A Fig) and comprised on average 99% of the OTUs assigned to phylum level (S2A Table).

The major classes of bacteria found in all treatments, in order of prevalence, were Betaproteobacteria (35% of total OTUs), Gammaproteobacteria (29%), Actinobacteria (14%), Alphaproteobacteria (9%), Cytophagia (5%), and Verrucomicrobia (1%). In the non-exposed animals, Cytophagia was the third most abundant group, contributing 8 to 36% throughout the experiment, whereas Actinobacteria contributed less than 2% on average (S4 Fig). Bacilli, Sphingobacteria and Bacteroidia were found together in about 3% of total reads assigned at class level (S2B Table, S5B Fig).

We found members of 62 orders in all treatments (S2C Table). Predominant orders included Burkholderiales (34%), Oceanospirillales (15%), Alteromonadales (10%), Rhizobiales (7%), Micrococcales (5%), and Cytophagales (5%), which was the second most represented order (16%) in the non-exposed animals (S4 Fig). The core gut microbiome were formed by these orders along with Propionibacteriales, Corynebacteriales, Pseudomonadales and Methylophilales (S5C Fig) representing almost 89% of the OTUs assigned at the order level.

Members of 101 families comprising 252 genera were identified as unique reads and assigned at the family and genus level. Across the treatments, Comamonadaceae (33%), *Halomonadaceae* (15%), *Shewanellaceae* (10%), and *Cytophagaceae* (5%) were the most common (S2E Table). In the non-exposed animals, *Comamonadaceae* (65%) and *Cytophagaceae* (17%) were the most common (S4 Fig).

When all treatments were considered, the most abundant genera were *Limnohabitans*, *Shewanella*, *Halomonas*, *Bosea*, and *Leadbetterella*. These genera contributed on average 71% (ranging from 57% to 81%) to the gut microbiota (S5E Fig). In the non-exposed animals, however, *Bosea* was not contributing to the core microbiome (S4 Fig).

### Effects of Ciprofloxacin on the core microbiome

**Fig 2:**
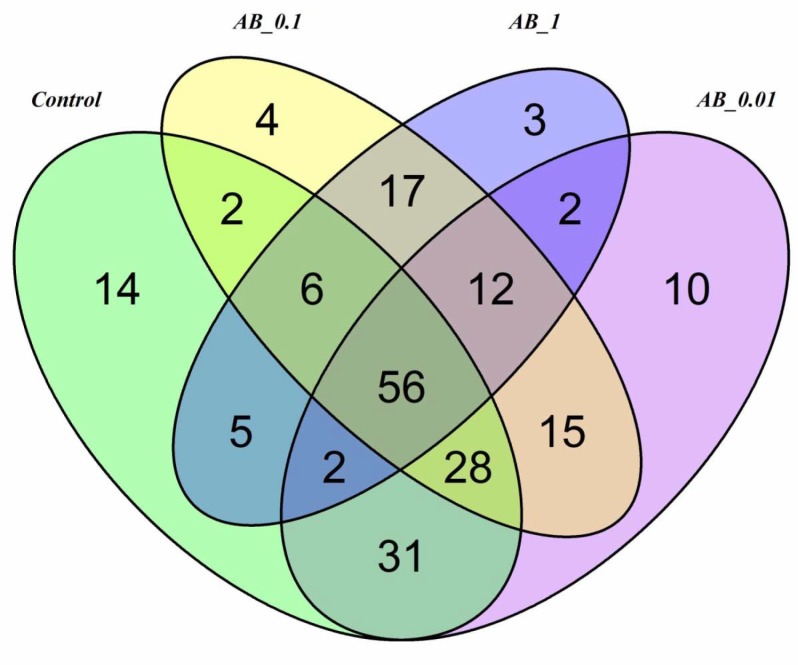
Venn diagram of shared and unique OTUs in the treatments exposed to Ciprofloxacin and in the control. Venn diagram was generated using the rarefied OTUs after applying low count filter of 4 reads with prevalence of 20% in each sample. Shared taxa present in all four groups (100% core threshold) were operationally defined as the core microbiome.

Using the selected filtering settings and pooling all samples collected over the course of the experiment, we identified 144, 156, 140, and 103 OTUs (207 unique OTUs in total) in the controls and the groups exposed to 0.01, 0.1 and 1 mgL^-1^ Ciprofloxacin, respectively. Among the four groups, 56 OTUs were shared (Fig 2), corresponding to 27% of all OTUs. Furthermore, 6 shared classes, 8 shared orders, 8 shared families, and 10 shared genera were identified (Fig 2; S5 Fig). These taxa can be regarded as the core microbiome of *Daphnia magna* gut.

### Effects of time and Ciprofloxacin on the microbiome diversity

**Fig 3:**
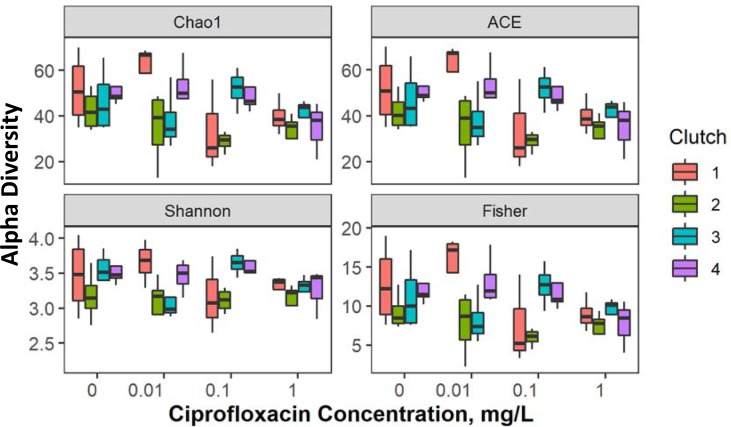
Alpha diversity indices (Chao1, ACE, Shannon-Weiner and Fisher’s alpha) for gut microbiota in *Daphnia magna*.

Diversity indices were calculated using rarefied OTU data for the samples analyzed during the experiment across the concentrations of Ciprofloxacin (mg L^-1^) tested (S3 Table). The diversity indices showed varying trends over time, with high initial diversity (up to the first clutch), a decrease observed at the time of the second clutch, following in some cases by an increasing trend toward the end of the experiment (Fig 3). The positive effect of time was significant for Fisher’s alpha, but not for Chao1, ACE and Shannon-Weiner indices (Table 2). For all indices except Shannon-Wiener, the negative effect of concentration was significant; it was also more profound than the time effect for Fisher’s alpha (Table 2).

**Table 2:** Effects of time and concentration on the diversity indices.

Diversity index	Factor	Estimate	Std. Error	*p* value
Fisher´s alpha	Concentration	-0.412471	0.120011	**0.0005**
	Time	0.032795	0.00987	**0.0008**
Chao1	Concentration	-0.20608	0.102056	**0.043**
	Time	0.005290	0.009492	0.577
ACE	Concentration	0.205	0.101	**0.043**
	Time	0.006	0.009	0.533
Shannon-Wiener	Concentration	-0.046	0.032	0.15
	Time	0.002	0.003	0.4

Diversity indices calculated using individual data rarefied to equal sequencing depth at treatment level. Effects of concentration and time on the diversity indices (Fisher´s alpha, Chao1, ACE and Shannon-Wiener) were evaluated using GLM with normal error structure and log-link. Interaction *time × concentration* were included in each model but omitted when found not significant. Significant *p* values are in bold face.

**Fig 4:**
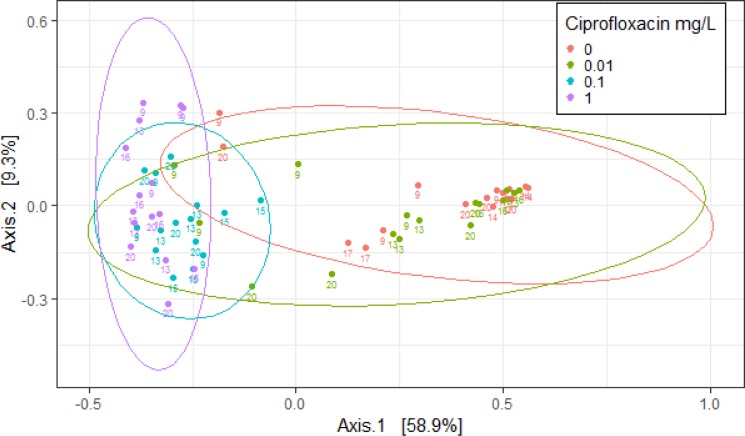
Principle Coordinate Analysis (PCoA) based on Bray Curtis dissimilarity metrics, showing the distance in the bacterial communities between the treatments.

According to the PCoA, the microbiomes of the daphnids exposed to 0.1 and 1 mgL^-1^ clustered closely together, which separated them from the control and the 0.01 mgL^-1^ treatment along the first PC axis (Fig 4). Once the multivariate homogeneity was confirmed (Betadisper: p >0.05; Table 3), a permutation test was performed which detected significant differences between the Ciprofloxacin treatments (PERMANOVA, *p* < 0.05).

**Table 3:** Analysis of multivariate homogeneity of group dispersions.

**Dispersion measures**					
	Control	0.01 mg/L	0.1 mg/L	1 mg/L	
Average distance to centroid	0.345	0.34	0.307	0.35	
Betadisper					
	**Df**	**Sum Sq**	**Mean Sq**	**Pseudo-F**	***P* value**
Treatment	3	0.017	0.006	1.194	0.317
Residuals	58	0.268	0.005		
**Betadisper pairwise comparisons**					
	Control	0.01 mg/L	0.1 mg/L	1 mg/L	
Control		0.823	0.135	0.877	
0.01 mg/L	0.822		0.204	0.673	
0.1 mg/L	0.111	0.202		0.062	
1 mg/L	0.864	0.696	0.068		

Multivariate homogeneity of variances was tested with Betadisper using samples originated from different treatments (Ciprofloxacin concentrations from 0.01 to 1 mg L^-1^) and control (0 mg L^-1^).

**Fig 5:**
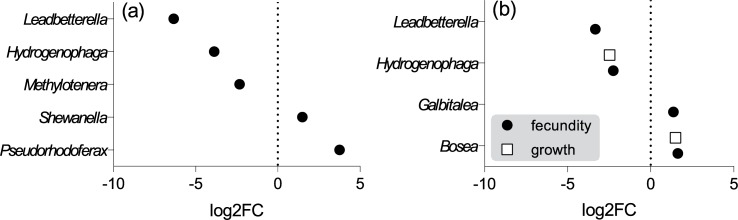
Differential abundance analysis of gut bacteria in *Daphnia magna* exposed to Ciprofloxacin. Bacterial genera significantly associated with (a) exposure to Ciprofloxacin; (b) high somatic growth and fecundity of the host observed during the experiment. The fold change (log2FC) and the associated statistics were determined using the *edgeR* package.

Further pairwise comparison demonstrated that all microbial communities were significantly different from each other (PERMANOVA: all pairwise comparisons *p* < 0.05; Table 4). Differential abundance analysis suggested that the most Ciprofloxacin sensitive bacteria were *Leadbetterella* (Bacteroidetes), *Hydrogenophaga* and *Methylotenera* both Betaproteobacteria. On the opposite end of the scale (most refractory) were *Pseudorhodoferax*, *Shewanella*, and *Halomonas* (Beta- and Gammaproteobacteria) as their abundance in the exposed animals had increased significantly following the antibiotic exposure (Fig 5A, S4 Table).

**Table 4:** Pairwise comparison of treatments using Bray-Curtis dissimilarity.

	**Df**	**SS**	**Pseudo-F**	**R**^**2**^		***P* value**
**Treatment**	3	2.584	6.884	0.263		0.001
**Residuals**	58	7.257		0.737		
**Total**	61	9.841		1		
**PERMANOVA pairwise comparisons (FDR corrected p)**						
	**Control**		**0.01 mg/L**		**0.1 mg/L**	
**0.01 mg/L**	0.01		-		-	
**0.1 mg/L**	0.001		0.001			
**1 mg/L**	0.001		0.001		0.001	

PERMANOVA output with Bray-Curtis dissimilarity testing differences between treatments at family level.

Communities grouped by Ciprofloxacin concentration and clutch number during the 21-d exposure. Data points indicate specific values for individual daphnids; the estimates were based on the rarefied OTUs libraries.

Color coding indicates treatments, i.e., concentration of Ciprofloxacin (0.01, 0.1, and 1 mg L^-1^) and control (0 mg L^-1^). The ellipsoids represent a 95% confidence interval (normal distribution) surrounding each group, and point labels indicate day of sampling. Plot shows the clear clustering of bacterial communities in the treatments exposed to the two highest concentrations of Ciprofloxacin (0.1 and 1 mg L^-1^) as well as between the communities in the controls and the lowest exposure concentration (0.01 mg L^-1^).

### Effects of Ciprofloxacin on antioxidant capacity in daphnids

**Fig 6:**
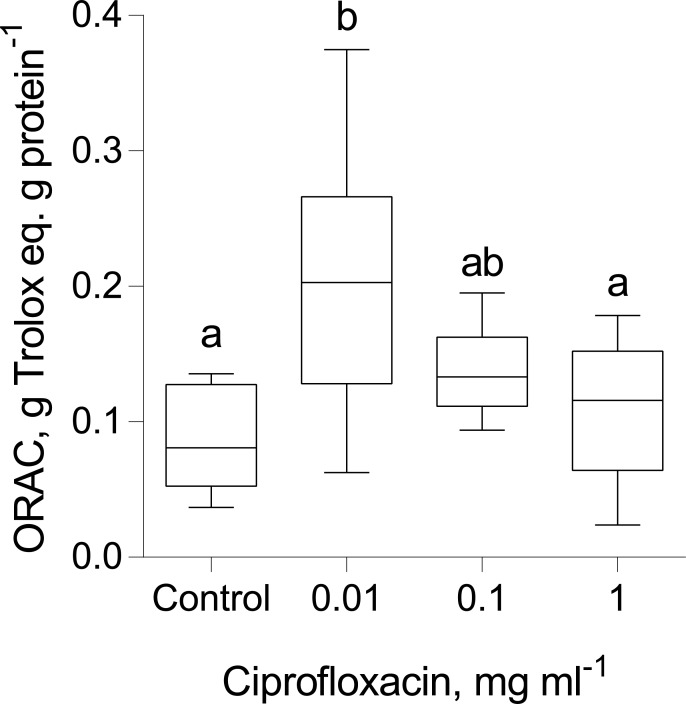
*Daphnia magna*: response of the total antioxidant capacity assayed as ORAC to the Ciprofloxacin concentration in the exposure. The individuals sampled after their fourth clutch were excluded for the ORAC (g Trolox eq. g protein^-1^) measurements, because some of them contained eggs in the brood chamber, which may affect the ORAC values. The non-matching letters indicate significant differences between the groups (Tukey's multiple comparisons test; p < 0.05). See Table 5 for the details on the statistical comparisons.

The total antioxidant capacity (ORAC, g Trolox eq. g protein^-1^) was significantly higher in the animals exposed to lower concentrations (0.01 and 0.1 mgL^-1^) of Ciprofloxacin (Fig 6, Table 5). Moreover, there was a significant positive relationship between the individual ORAC values and body length (GLM; Wald stat. = 5.83, p < 0.02; Table 5) across the treatments and time points.

**Table 5:** Effects of exposure (Ciprofloxacin, mg mL^-1^) on the antioxidant capacity in *Daphnia magna*.

**ANOVA results**	**SS**	**DF**	**MS**	**F (DFn, DFd)**	***p* value**
Treatment (concentration)	0.06387	3	0.02129	F (3, 32) = 5.969	0.0024
Residual	0.1141	32	0.003567		
Total	0.178	35			
**Tukey's multiple comparisons test**					
**Treatments, pair-wise**	**Mean Difference**			**95% CI of difference**	**Summary**
Control vs. 0.01	-0.1116			-0.1879 to -0.03537	**
Control vs. 0.1	-0.04873			-0.1250 to 0.02755	ns
Control vs. 1	-0.02017			-0.09645 to 0.05610	ns
0.01 vs. 0.1	0.06291			-0.01336 to 0.1392	ns
0.01 vs. 1	0.09147			0.01520 to 0.1677	*
0.1 vs. 1	0.02856			-0.04772 to 0.1048	ns

ANOVA and Tukey's multiple comparisons for the overall effect on the antioxidant capacity (ORAC values); p < 0.01: **, p < 0.05: *; and p > 0.05: ns. The individuals sampled at the termination of the experiment were excluded, because some daphnids contained eggs in the brood chamber. As the reference group, we used the daphnids exposed to the highest concentration. See also Fig 6.

### Linkages between gut microbiome, antioxidant capacity and life-history traits

All diversity indices correlated negatively with fecundity, while only Fisher’s alpha had a positive correlation with body size (S6 Table). Moreover, across the treatments, the correlations between the diversity indices and ORAC values were weakly negative and marginally significant for Chao1, Fisher’s alpha, and ACE (S7 Table).

The differential abundance analysis indicated that genera *Bosea* and *Hydrogenophaga* were more abundant in the daphnids with high and low somatic growth, respectively (S8 Table, Fig 5B). Moreover, *Bosea* and *Galbitalea* were significantly more abundant in the more fecund daphnids, whereas abundances of *Leadbetterella* and *Hydrogenophaga* in these individuals were significantly lower (S8 Table, Fig 5B). Thus, *Bosea* and *Hydrogenophaga* were consistently associated with high and low growth phenotypes, respectively.

## Discussion

The intestinal microbiome plays an essential role in regulating many aspects of host physiology, and its disruption through antibiotic exposure has been implicated in microbiota-mediated consequences on host fitness. We examined effects of chronic exposure to antibiotics on *Daphnia magna* gut microbiota in concert with fitness-related responses of the host. As hypothesized, the exposure to Ciprofloxacin resulted in profound changes in the microbiome and a reduced microbial diversity at all concentrations tested (0.01 to 1 mg L^-1^). Surprisingly, no negative effects on daphnid antioxidant levels, fitness and mortality were observed. Moreover, decrease in microbial diversity coincided with increased antioxidant capacity, individual growth and host reproduction and, as a result, significantly higher population growth in the animals exposed to Ciprofloxacin. Thus, the hypothesized positive correlation between microbiome diversity and host performance was not observed. These findings imply that reliance on shifts in taxonomic composition of bacterial community generates an incomplete picture of the functional effect of antibiotic intervention in a non-target eukaryote. A full mechanistic understanding will require further study of the specific functional relationships between the host and its core microbiome, and the integration of metabolomic and phenotypic data. Moreover, in case of antibiotic-mediated intervention, we need to disentangle direct effects of the exposure on host physiology.

### Core microbiome of *Daphnia magna*

Proteobacteria, Actinobacteria and Bacteroidetes comprise a core microbiome of the *Daphnia magna* intestine. Most taxa (or their close relatives) identified in this study as a part of core microbiome have previously been reported in *Daphnia* [20,21,45]. The Comamonadaceae family of Burkholderiales have been shown to be the most abundant family in *Daphnia* gut microbiota [20,46] and were most prevalent in our test animals. Other highly abundant taxa were Gammaproteobacteria, orders Oceanospirillales and Alteromonadales, and the families *Nocardioidaceae*, *Microbacteriaceae*, and *Moraxellaceae* [12,21].

On the genus level, greater differences between the earlier reports on microbiota composition in *Daphnia* and our dataset were evident. In addition to *Limnohabitans*, other identified taxa were *Pseudorhodoferax* and *Hydrogenophaga* (Burkholderiales) but not the previously reported *Bordetella*, *Cupriavidus* [21] *Ideonella* and *Leptothrix* [20]. Also, *Enhydrobacter* was the dominant genus of *Moraxellaceae* in our study (S2E Table), while *Acinetobacter* was reported in other studies [12,20]. *Methylibium* was only found in the animals that were exposed to 0.01 mg L^-1^ of Ciprofloxacin and not in the controls, suggesting that this genus is relatively rare if ordinarily present. Together, our findings suggest a relatively stable bacterial composition in the *Daphnia* gut at the higher taxonomic level, suggestive of functional redundancy in the interactions between the daphnids and their microbiota.

### Effects of Ciprofloxacin on the *Daphnia* gut microbiome

Ciprofloxacin exposure significantly altered the microbiome, with a decrease or even the disappearance of many taxa by the end of the experiment at the lowest exposure concentration and within the first week at higher concentrations (S2 Table). Although Fisher’s alpha diversity decreased with both Ciprofloxacin concentration and exposure time (Fig 3), only the concentration effect was significant for Chao 1 and ACE; none of the effects were significant for Shannon-Weiner index (S3 Table). The G+ bacteria, mostly *Actinobacteria* and *Firmicutes*, were better equipped to withstand Ciprofloxacin exposure as their relative abundance increased with drug concentration, while the G- bacteria had divergent responses (S7 Fig). For example, *Hydrogenophaga* and *Pseudorhodoferax*, both belonging to the G- genus *Burkholderiales*, had clearly opposite responses, decreasing and increasing, respectively, with increasing concentration. This is in line with earlier studies that demonstrated higher susceptibility to Ciprofloxacin among the G- bacteria as compared with co-occurring G+ species [26]. This is supported by the typically low minimum inhibitory concentrations, MICs, estimated for Alphaproteobacteria, such as *Escherichia/Shigella*, (commonly in the low μM range) as compared with that for many Firmicutes, which are usually in the mM range.

At higher concentrations of Ciprofloxacin, several genera of the core microbiome declined to non-detectable levels. In particular, the *Limnohabitans* genus was replaced by *Halomonas* and *Shewanella*, whose relative abundances increased with drug concentration (S2E Table). *Shewanella* is a known acid producer [47] and at higher densities it may alter the pH balance in the gut microenvironment. This would suppress growth of *Limnohabitans* that prefers neutral and alkaline conditions [48]. Such community-level effects mediated by competition between the microbial consortia probably play a significant role in the dynamics of specific bacterial taxa as a result of the exposure to antibiotics.

### Effects of Ciprofloxacin on life history traits and antioxidant levels in *Daphnia*

Studies on aposymbiotic daphnids showed that disruption in gut microbiota, either by drugs or a diet, had adverse effects on nutrition [11], immunity, growth [9], fecundity [22], and longevity [49]. The effects that we observed, however, were most prominent at low antibiotic concentrations, which are below the typical MICs for bacteria [30]. Despite the Ciprofloxacin-induced shifts in the microbiome diversity and composition, ORAC levels, growth and reproduction in the daphnids were similar or even significantly higher than in the controls. The discrepancy between the microbiome and the organism-level responses may result from differential susceptibility of various microbes to the broad-spectrum Ciprofloxacin and additional variability related to induction of the SOS response pathways in different taxa.

The mismatch between microbiome change and host response suggests that other drivers, such as a direct effect of Ciprofloxacin on the host, might have been were involved, leading to the observed effects on growth and reproduction. In line with this, a biphasic dose-response to Ciprofloxacin observed in human fibroblast cells, manifesting as increased cell proliferation and viability when compared to non-exposed controls [50]. In *Daphnia magna*, the reproduction response to Ciprofloxacin was also biphasic, with stimulatory effects at concentrations below 5 mg L^-1^ [51]. This is in line with the positive response induced by the test concentrations utilized in our study (0.01–1 mg L^-1^). In mice, Ciprofloxacin has also been shown to improve survival by enhancing immune efficiency via stimulating cytokine production [52]. In addition, several *in vitro* and *in vivo* studies using animal and tissue models have revealed that fluoroquinolones, such as Ciprofloxacin, induce oxidative stress via reactive oxygen species (ROS) production, in a dose- and time-dependent manner [52,53]. Measurable ROS production was observed following exposure to Ciprofloxacin at concentrations as low as 0.025 mM [53], which is within the concentration range used in our study. At low levels of such pro-oxidative exposure, the additional production and/or activity of the endogenous antioxidant enzymes and low-molecular weight antioxidants to remove the continuously generated free radicals would increase [54]. In the daphnids exposed to the lowest Ciprofloxacin concentration, a significant increase in ORAC levels (S6 Fig) suggests that exposure had direct stimulatory effects on the antioxidant production. Moreover, we observed a positive correlation between the ORAC levels and animal body size across the treatments indicating a possible primary mechanism behind the observed effects being a hormetic shifting of redox environment by the pro-oxidative Ciprofloxacin, antioxidant response and the resulting beneficial effects on growth. Such effects are in agreement with a concept of physiological conditional hormesis [55] and suggest a possible mechanism for the direct response of *Daphnia magna* to Ciprofloxacin exposure at environmentally relevant concentrations. An important caveat is that hormesis, also shown to occur in several microbes’ response to quinolones and fluoroquinolones (the so-called paradoxical effect) [56] might be universal and thus Ciprofloxacin may be a suboptimal choice for the uncomplicated study on microbiome involvement in dose-response relationships with the host. As a model system to experimentally disentangle drug effects on the eukaryotic host from those on its microbiome, gnotobiotic daphnids can be used [3,9].

### Microbiome-fitness relationships

Although elevated growth and reproduction were associated with some bacterial taxa, there was no clear signal for the involvement of the gut microbiome in the high-growth phenotype. This is suggestive of a redundancy in host-microbiome function, i.e., microbes can be exchanged with little or no penalty for fitness-related endpoints. Moreover, as mechanisms governing most observed associations are not well understood, definitive conclusion of direct effects by specific microbes is intuitively discouraged. In particular, several taxa (*Bosea* and *Shewanella*) significantly associated with fitness-related variables have been shown to be highly resistant to Ciprofloxacin [57,58]. The selection, even acting directly on the polymicrobial community, does so differentially. Although the effect may be due to absolute numbers of microbes, the cumulative physiological and metabolic state may matter more. In line with this, the relative abundance of those genera that were associated with higher fecundity and growth barely comprise 5% of the organism’s microbiome (S2 Table), suggesting that sheer abundance was unlikely to be the primary factor driving the host fitness.

It is a common view that strains capable of supplying essential elements for reproduction and growth would benefit the host. For example, the key components of *Daphnia* gut microbiota, *Limnohabitans*, *Aeromonas* and methanotrophic bacteria [59], have been linked to acquisition of essential amino acids [60,61], polyunsaturated fatty acids (PUFA) and sterols [62] that positively affect *Daphnia* growth and reproduction [9,61]. Surprisingly, none of these taxa were associated with elevated growth and fecundity in our study. This also speaks for functional redundancy although additional studies would be required to show this. At the genus level, only *Bosea* and *Galbitalea* had significantly positive association with *Daphnia* growth and fecundity, whereas the association for *Leadbetterella* and *Hydrogenophaga*, which are commonly found in *Daphnia* [63], was negative. The *Bradyrhizobiaceae* (*Bosea*) and *Microbacteriaceae* (*Galbitalea*) are bio-degraders capable of producing hydrolytic enzymes such as chitinase, cellulase, glucanase, protease, etc. [57,64]. Therefore, an increased network density and number of degradation pathways may provide essential nutrients from more available substrates [65], which may contribute to the observed positive association between the relative abundance of these taxa with fecundity and host fitness. Regardless of the mechanisms underlying their increased relative abundance, resistance, or at the very least, refractoriness to Ciprofloxacin cannot be ignored. Such effects would be evident in perturbed outcome of inter- and intra-species competition and illustrates one of the difficulties facing studies of the host-microbiome interactions.

## Supporting information

S1 File R script for calculation of population growth rate according to Euler-Lotka equation (Eq 2). (TXT)

S1 Table Population growth rate analysis. Population growth rate (*r*) of *Daphnia magna* in the control and Ciprofloxacin exposure (0.01–1 mg L^-1^) and the corresponding 95-% confidence interval estimated by bootstrapping. Asterisk indicates significant difference from the control; when the confidence intervals were not overlapping, the difference was considered significant.
(XLSX)

S2 Table Overview of relative abundances of the dominant bacteria across the treatments. Relative contributions of the ten most common bacterial taxa in the gut microbiota of *Daphnia magna* exposed to Ciprofloxacin (0.01. 0.1. and 1 mg L^-1^) and in control (0 mg L^-1^) as well as the average relative abundance for all treatments.
(XLSX)

S3 Table Diversity indices used in the alpha diversity analysis for each sample. Diversity indices were calculated using rarefied OTU data. Information is provided for the samples analyzed during the experiment across the concentrations of Ciprofloxacin (mg L^-1^) tested (Concentration 0 is the control group) and variables representing the time of exposure as a clutch number, 1 to 4, and day of experiment corresponding to the sampling event.
(XLSX)

S4 Table Differential abundance of individual genera representing taxa-specific responses to Ciprofloxacin exposure. The positive log2FC values indicate increased relative abundance in the exposed daphnids compared to the controls. Significance presented at false discovery rate of 5% (FDR<0.05) estimated by *edgeR* package.
(XLSX)

S5 Table Relationship between the total antioxidant capacity assayed as ORAC and daphnid body length. Generalized linear model output linking antioxidant capacity assayed by ORAC to daphnid body length across the treatments and time points. Normal error structure and log-link function were applied. The animals collected at the termination of the experiment were excluded, because they had eggs in the brood chambers, which may affect the ORAC values.
(XLSX)

S6 Table Spearman correlations between the diversity indices and fitness-related parameters. The diversity indices used in the alpha diversity analysis were correlated to variables representing growth and reproduction: Body length of the daphniids at the time of sampling for 16S rRNA gene sequencing, Fecundity rank used in the differential abundance analysis (scored 0 to 3), and Size-specific fecundity calculated using Brood size and Body length. Significant correlations (p < 0.05) are in red.
(XLSX)

S7 Table Spearman correlation coefficients between ORAC levels and diversity indices for gut microbiome in *Daphnia magna*. The ORAC values and diversity indices were assayed in individual daphnids (*n* = 62). All treatments and time points were included in this analysis. Marginally significant *p* values are in italics.
(XLSX)

S8 Table Differential abundance analysis of individual genera estimated by *edgeR*-function and testing associations between the microbiome and host fitness parameters, fecundity and growth. The genera positively associated with high growth assayed as increase in body length or fecundity assayed as brood size of *D*. *magna* have positive log2FC values. All values reported are significant at false discovery rate of 1%. (FDR<0.01). See also Fig 5.
(XLSX)

S1 Fig Kaplan-Meier curves and estimates of survival data. Survival of *Daphnia magna* exposed to Ciprofloxacin (0.01, 0.1 and 1 mg L^-1^) and in the control during the 21-d exposure.
(PDF)

S2 Fig Neonate production in the exposed and non-exposed animals. Reproduction of *Daphnia magna* (brood size and time of reproduction) during a 21-d exposure to Ciprofloxacin (0.01, 0.1, and 1 mg L^-1^) and the control. Note that the last clutch was estimated using both the offspring released and the embryos in the brood chamber at the termination of the experiment.
(PDF)

S3 Fig Rarefaction curves of gut microbiota OTUs in *Daphnia magna*. Rarefaction curves show the cumulative number of unique OTUs as a function of sample size (number of reads for the 16s rRNA gene) for all individuals sampled in different treatments (Ciprofloxacin concentration, mgL^-1^) and control during the experiment. Colors denote the clutch number.
(PDF)

S4 Fig Relative abundance of bacterial taxa in the microbiome of *Daphnia magna* from the controls. The abundances are shown for the different taxonomy ranks: (a) Phylum, (b) Class, (c) Order, (d) Family, and (e) Genus. Along the vertical axis, the data are grouped by the clutch, 1 to 4, produced during the experiment.
(PDF)

S5 Fig The heatmap of the core microbiome in *Daphnia magna*. The heatmap of the core microbiome in *Daphnia magna* collected during the experiment across different taxonomic categories: (a) Phylum (b) class, (c) order, (d) family, and (e) genera.
(PDF)

S6 Fig Variation in ORAC levels measured in *Daphnia magna* from different treatments. The total antioxidant capacity (ORAC, g Trolox eq./ g protein) was assayed in individual daphnids during the course of the experiment. The data are shown for the control (Concentration 0 mg L^-1^) and each treatment (Ciprofloxacin concentration: 0.01, 0.1 and 1 mg L^-1^). The regression line and the 95%-confidence interval are shown to indicate the overall direction of change over time in different treatments.
(PDF)

S7 Fig Changes in relative abundance of Gram-positive (G+) and Gram-negative (G-) bacteria in response to Ciprofloxacin exposure. Fold-change of G- and G+ bacteria in gut microbiota of *D*. *magna* exposed to Ciprofloxacin (0 to 1 mg/L). For G+ bacteria at the order level, mostly increase in response to Ciprofloxacin was observed as shown for, for example, Actinobacteria (a) and Firmicutes (b). For G- bacteria, the responses were more divergent. For example, responses of Pseudorhodoferax (c) and Hydrogenophaga (d) families belonging to the same order *Burkholderiales* were the opposite.
(PDF)

## References

[1] O’Hara AM, Shanahan F. The gut flora as a forgotten organ. EMBO Rep. 2006;7: 688–693. 10.1038/sj.embor.7400731 16819463 PMC1500832

[2] Willing BP, Russell SL, Finlay BB. Shifting the balance: antibiotic effects on host–microbiota mutualism. Nat Rev Microbiol. 2011;9: 233–243. 10.1038/nrmicro2536 21358670

[3] Rosenfeld CS. Gut Dysbiosis in Animals Due to Environmental Chemical Exposures. Front Cell Infect Microbiol. 2017;7 10.3389/fcimb.2017.00396 28936425 PMC5596107

[4] Lee W-J, Hase K. Gut microbiota-generated metabolites in animal health and disease. Nat Chem Biol. 2014;10: 416–424. 10.1038/nchembio.1535 24838170

[5] McFall-Ngai M, Hadfield MG, Bosch TC, Carey HV, Domazet-Lošo T, Douglas AE, et al Animals in a bacterial world, a new imperative for the life sciences. Proc Natl Acad Sci. 2013;110: 3229–3236. 10.1073/pnas.1218525110 23391737 PMC3587249

[6] Kamada N, Chen GY, Inohara N, Núñez G. Control of Pathogens and Pathobionts by the Gut Microbiota. Nat Immunol. 2013;14: 685–690. 10.1038/ni.2608 23778796 PMC4083503

[7] Cherrington CA, Hinton M, Pearson GR, Chopra I. Short-chain organic acids at ph 5.0 kill *Escherichia coli* and *Salmonella* spp. without causing membrane perturbation. J Appl Bacteriol. 1991;70: 161–165. 10.1111/j.1365-2672.1991.tb04442.x 1902205

[8] Shin R, Park JM, An J-M, Paek K-H. Ectopic Expression of Tsi1 in Transgenic Hot Pepper Plants Enhances Host Resistance to Viral, Bacterial, and Oomycete Pathogens. Mol Plant Microbe Interact. 2002;15: 983–989. 10.1094/MPMI.2002.15.10.983 12437295

[9] Sison-Mangus MP, Mushegian AA, Ebert D. Water fleas require microbiota for survival, growth and reproduction. ISME J. 2015;9: 59–67. 10.1038/ismej.2014.116 25026374 PMC4274426

[10] Edlund A, Ek K, Breitholtz M, Gorokhova E. Antibiotic-Induced Change of Bacterial Communities Associated with the Copepod *Nitocra spinipes*. PLoS ONE. 2012;7: e33107 10.1371/journal.pone.0033107 22427962 PMC3299745

[11] Gorokhova E, Rivetti C, Furuhagen S, Edlund A, Ek K, Breitholtz M. Bacteria-Mediated Effects of Antibiotics on Daphnia Nutrition. Environ Sci Technol. 2015;49: 5779–5787. 10.1021/acs.est.5b00833 25850437

[12] Callens M, Watanabe H, Kato Y, Miura J, Decaestecker E. Microbiota inoculum composition affects holobiont assembly and host growth in Daphnia. Microbiome. 2018;6: 56 10.1186/s40168-018-0444-1 29566771 PMC5863831

[13] Gyuraszova M, Kovalcikova A, Gardlik R. Association between oxidative status and the composition of intestinal microbiota along the gastrointestinal tract. Med Hypotheses. 2017;103: 81–85. 10.1016/j.mehy.2017.04.011 28571818

[14] Dietrich S, Ploessl F, Bracher F, Laforsch C. Single and combined toxicity of pharmaceuticals at environmentally relevant concentrations in *Daphnia magna*–A multigenerational study. Chemosphere. 2010;79: 60–66. 10.1016/j.chemosphere.2009.12.069 20116828

[15] Brennan SJ, Brougham CA, Roche JJ, Fogarty AM. Multi-generational effects of four selected environmental oestrogens on *Daphnia magna*. Chemosphere. 2006;64: 49–55. 10.1016/j.chemosphere.2005.11.046 16405951

[16] Wollenberger L, Halling-Sørensen B, Kusk KO. Acute and chronic toxicity of veterinary antibiotics to *Daphnia magna*. Chemosphere. 2000;40: 723–730. 10.1016/s0045-6535(99)00443-9 10705550

[17] Tanaka Y, Nakanishi J. Chronic effects of p-nonylphenol on survival and reproduction of *Daphnia galeata*: Multigenerational life table experiment. Env Toxicol. 2002;17: 487–492. 10.1002/tox.10083 12242680

[18] Harris KDM, Bartlett NJ, Lloyd VK. Daphnia as an Emerging Epigenetic Model Organism. Genet Res Int. 2012;2012 10.1155/2012/147892 22567376 PMC3335723

[19] Stollewerk A. The water flea Daphnia—a “new” model system for ecology and evolution? J Biol. 2010;9: 21 10.1186/jbiol212 20478012 PMC2871515

[20] Freese HM, Schink B. Composition and Stability of the Microbial Community inside the Digestive Tract of the Aquatic Crustacean *Daphnia magna*. Microb Ecol. 2011;62: 882 10.1007/s00248-011-9886-8 21667195

[21] Qi W, Nong G, Preston JF, Ben-Ami F, Ebert D. Comparative metagenomics of Daphnia symbionts. BMC Genomics. 2009;10: 172 10.1186/1471-2164-10-172 19383155 PMC2678164

[22] Peerakietkhajorn S, Kato Y, Kasalický V, Matsuura T, Watanabe H. Betaproteobacteria Limnohabitans strains increase fecundity in the crustacean *Daphnia magna*: symbiotic relationship between major bacterioplankton and zooplankton in freshwater ecosystem. Environ Microbiol. 2016;18: 2366–2374. 10.1111/1462-2920.12919 26014379

[23] Huang D-J, Hou J-H, Kuo T-F, Lai H-T. Toxicity of the veterinary sulfonamide antibiotic sulfamonomethoxine to five aquatic organisms. Environ Toxicol Pharmacol. 2014;38: 874–880. 10.1016/j.etap.2014.09.006 25461547

[24] De Liguoro M, Fioretto B, Poltronieri C, Gallina G. The toxicity of sulfamethazine to Daphnia magna and its additivity to other veterinary sulfonamides and trimethoprim. Chemosphere. 2009;75: 1519–1524. 10.1016/j.chemosphere.2009.02.002 19269673

[25] Test No. 211: Daphnia magna Reproduction Test—en—OECD [Internet]. [cited 25 Sep 2019]. Available: https://www.oecd.org/chemicalsafety/test-no-211-daphnia-magna-reproduction-test-9789264185203-en.htm

[26] LeBel M. Ciprofloxacin: Chemistry, Mechanism of Action, Resistance, Antimicrobial Spectrum, Pharmacokinetics, Clinical Trials, and Adverse Reactions. Pharmacother J Hum Pharmacol Drug Ther. 1988;8: 3–30. 10.1002/j.1875-9114.1988.tb04058.x 2836821

[27] Castiglioni S, Bagnati R, Fanelli R, Pomati F, Calamari D, Zuccato E. Removal of Pharmaceuticals in Sewage Treatment Plants in Italy. Environ Sci Technol. 2006;40: 357–363. 10.1021/es050991m 16433372

[28] Lien LTQ, Hoa NQ, Chuc NTK, Thoa NTM, Phuc HD, Diwan V, et al Antibiotics in Wastewater of a Rural and an Urban Hospital before and after Wastewater Treatment, and the Relationship with Antibiotic Use—A One Year Study from Vietnam. Int J Environ Res Public Health. 2016;13 10.3390/ijerph13060588 27314366 PMC4924045

[29] Robinson AA, Belden JB, Lydy MJ. Toxicity of fluoroquinolone antibiotics to aquatic organisms. Environ Toxicol Chem. 2005;24: 423–430. 10.1897/04-210r.1 15720004

[30] Grillon A, Schramm F, Kleinberg M, Jehl F. Comparative Activity of Ciprofloxacin, Levofloxacin and Moxifloxacin against *Klebsiella pneumoniae*, *Pseudomonas aeruginosa* and *Stenotrophomonas maltophilia* Assessed by Minimum Inhibitory Concentrations and Time-Kill Studies. Nguyen MH, editor. PLOS ONE. 2016;11: e0156690 10.1371/journal.pone.0156690 27257956 PMC4892626

[31] Collins TJ. ImageJ for microscopy. BioTechniques. 2007;43: S25–S30. 10.2144/000112517 17936939

[32] Straughan DJ, Lehman N. Genetic differentiation among Oregon lake populations of the *Daphnia pulex* species complex. J Hered. 2000;91: 8–17. 10.1093/jhered/91.1.8 10739118

[33] Logares R., & Feng X. Quant-iT PicoGreen Assay. Quant-IT PicoGreen Assay. 2010;

[34] Callahan BJ, McMurdie PJ, Rosen MJ, Han AW, Johnson AJA, Holmes SP. DADA2: High resolution sample inference from Illumina amplicon data. Nat Methods. 2016;13: 581–583. 10.1038/nmeth.3869 27214047 PMC4927377

[35] Team RC. R: A language and environment for statistical computing Vienna, Austria: R Foundation for Statistical Computing; 2017 ISBN3-900051-07-0 https://www.R-project.org; 2017.

[36] McMurdie PJ, Holmes S. phyloseq: An R Package for Reproducible Interactive Analysis and Graphics of Microbiome Census Data. PLOS ONE. 2013;8: e61217 10.1371/journal.pone.0061217 23630581 PMC3632530

[37] Ou B, Hampsch-Woodill M, Prior RL. Development and Validation of an Improved Oxygen Radical Absorbance Capacity Assay Using Fluorescein as the Fluorescent Probe. J Agric Food Chem. 2001;49: 4619–4626. 10.1021/jf010586o 11599998

[38] Furuhagen S, Liewenborg B, Breitholtz M, Gorokhova E. Feeding Activity and Xenobiotics Modulate Oxidative Status in *Daphnia magna*: Implications for Ecotoxicological Testing. Environ Sci Technol. 2014;48: 12886–12892. 10.1021/es5044722 25247638

[39] Kaplan EL, Meier P. Nonparametric Estimation from Incomplete Observations Breakthroughs in Statistics. Springer, New York, NY; 1992 pp. 319–337. 10.1007/978-1-4612-4380-9_25

[40] Borgan Ø. Modeling Survival Data: Extending the Cox Model Terry M. Therneau and Patricia M. Grambsch, Springer-Verlag, New York, 2000 No. of pages: xiii + 350. Price: $69.95. ISBN 0-387-98784-3. Stat Med. 2001;20: 2053–2054. 10.1002/sim.956

[41] Chao A, Jost L. Coverage-based rarefaction and extrapolation: standardizing samples by completeness rather than size. Ecology. 2012;93: 2533–2547. 10.1890/11-1952.1 23431585

[42] Gower JC. Some distance properties of latent root and vector methods used in multivariate analysis. Biometrika. 1966;53: 325–338. 10.1093/biomet/53.3-4.325

[43] Oksanen J, Blanchet FG, Friendly M, Kindt R, Legendre P, McGlinn D, et al vegan: Community Ecology Package [Internet]. 2018 Available: https://CRAN.R-project.org/package = vegan

[44] McCarthy DJ, Chen Y, Smyth GK. Differential expression analysis of multifactor RNA-Seq experiments with respect to biological variation. Nucleic Acids Res. 2012;40: 4288–4297. 10.1093/nar/gks042 22287627 PMC3378882

[45] Eckert EM, Pernthaler J. Bacterial epibionts of Daphnia: a potential route for the transfer of dissolved organic carbon in freshwater food webs. ISME J. 2014;8: 1808–1819. 10.1038/ismej.2014.39 24694716 PMC4139729

[46] Kasalický V, Jezbera J, Šimek K, Hahn MW. *Limnohabitans planktonicus* sp. nov., and *Limnohabitans parvus* sp. nov., two novel planktonic Betaproteobacteria isolated from a freshwater reservoir. Int J Syst Evol Microbiol. 2010;60: 2710–2714. 10.1099/ijs.0.018952-0 20061501 PMC3091486

[47] Bowman JP. Genus XIII. Shewanella MacDonell and Colwell 1986, 355VP (Effective publication: MacDonell and Colwell 1985, 180). 2005; Available: http://ecite.utas.edu.au/39287

[48] Jezbera J, Jezberová J, Koll U, Horňák K, Šimek K, Hahn MW. Contrasting trends in distribution of four major planktonic betaproteobacterial groups along a pH gradient of epilimnia of 72 freshwater habitats. FEMS Microbiol Ecol. 2012;81: 467–479. 10.1111/j.1574-6941.2012.01372.x 22452571 PMC4268498

[49] Peerakietkhajorn S, Tsukada K, Kato Y, Matsuura T, Watanabe H. Symbiotic bacteria contribute to increasing the population size of a freshwater crustacean, *Daphnia magna*. Environ Microbiol Rep. 2015;7: 364–372. 10.1111/1758-2229.12260 25534397

[50] Hincal F, Gürbay A, Favier A. Biphasic Response of Ciprofloxacin in Human Fibroblast Cell Cultures. Nonlinearity Biol Toxicol Med. 2003;1: 481–492. 10.1080/15401420390271083 19330132 PMC2656119

[51] Dalla Bona M, Zounková R, Merlanti R, Blaha L, De Liguoro M. Effects of enrofloxacin, ciprofloxacin, and trimethoprim on two generations of *Daphnia magna*. Ecotoxicol Environ Saf. 2015;113: 152–158. 10.1016/j.ecoenv.2014.11.018 25497771

[52] Purswani MU, Eckert SJ, Arora HK, Noel GJ. Effect of ciprofloxacin on lethal and sublethal challenge with endotoxin and on early cytokine responses in a murine in vivo model. J Antimicrob Chemother. 2002;50: 51–58. 10.1093/jac/dkf091 12096006

[53] Gürbay A, Hıncal F. Ciprofloxacin‐Induced Glutathione Redox Status Alterations in Rat Tissues. Drug Chem Toxicol. 2004;27: 233–242. 10.1081/dct-120037504 15478945

[54] Gürbay A, Gonthier B, Daveloose D, Favier A, Hincal F. Microsomal metabolism of ciprofloxacin generates free radicals. Free Radic Biol Med. 2001;30: 1118–1121. 10.1016/s0891-5849(01)00508-1 11369501

[55] Oliveira MF, Geihs MA, França TFA, Moreira DC, Hermes-Lima M. Is “Preparation for Oxidative Stress” a Case of Physiological Conditioning Hormesis? Front Physiol. 2018;9 10.3389/fphys.2018.00945 30116197 PMC6082956

[56] Crumplin GC, Smith JT. Nalidixic Acid: an Antibacterial Paradox. Antimicrob Agents Chemother. 1975;8: 251–261. 10.1128/aac.8.3.251 1101818 PMC429302

[57] Ouattara AS, Assih EA, Thierry S, Cayol J-L, Labat M, Monroy O, et al *Bosea minatitlanensis* sp. nov., a strictly aerobic bacterium isolated from an anaerobic digester. Int J Syst Evol Microbiol. 2003;53: 1247–1251. 10.1099/ijs.0.02540-0 13130002

[58] Yan L, Liu D, Wang X-H, Wang Y, Zhang B, Wang M, et al Bacterial plasmid-mediated quinolone resistance genes in aquatic environments in China. Sci Rep. 2017;7 10.1038/srep40610 28094345 PMC5240147

[59] Peerakietkhajorn S, Tsukada K, Kato Y, Matsuura T, Watanabe H. Symbiotic bacteria contribute to increasing the population size of a freshwater crustacean, *Daphnia magna*. Environ Microbiol Rep. 2015;7: 364–372. 10.1111/1758-2229.12260 25534397

[60] Fink P, Pflitsch C, Marin K. Dietary Essential Amino Acids Affect the Reproduction of the Keystone Herbivore *Daphnia pulex*. PLOS ONE. 2011;6: e28498 10.1371/journal.pone.0028498 22163027 PMC3230618

[61] Taipale SJ, Brett MT, Pulkkinen K, Kainz MJ. The influence of bacteria-dominated diets on *Daphnia magna* somatic growth, reproduction, and lipid composition. FEMS Microbiol Ecol. 2012;82: 50–62. 10.1111/j.1574-6941.2012.01406.x 22564190

[62] Wacker A, Elert E von. Polyunsaturated Fatty Acids: Evidence for Non-Substitutable Biochemical Resources in *Daphnia Galeata*. Ecology. 82: 2507–2520. 10.1890/0012-9658(2001)082[2507:PFAEFN]2.0.CO;2

[63] Sison-Mangus MP, Metzger CMJA, Ebert D. Host genotype-specific microbiota do not influence the susceptibility of D. magna to a bacterial pathogen. Sci Rep. 2018;8: 9407 10.1038/s41598-018-27681-x 29925845 PMC6010447

[64] Shivlata L, Satyanarayana T. Thermophilic and alkaliphilic Actinobacteria: biology and potential applications. Front Microbiol. 2015;6 10.3389/fmicb.2015.01014 26441937 PMC4585250

[65] Chater KF, Biró S, Lee KJ, Palmer T, Schrempf H. The complex extracellular biology of *Streptomyces*. FEMS Microbiol Rev. 2010;34: 171–198. 10.1111/j.1574-6976.2009.00206.x 20088961

