# Supplementary material for: Disparate effects of antibiotic-induced microbiome change and enhanced fitness in Daphnia magna
Source: PLoS One. 2020 Jan 3;15(1):e0214833. doi: 10.1371/journal.pone.0214833 (PMC6941804; doi:10.1371/journal.pone.0214833)

Supporting Information

Disparate effects of antibiotic-induced microbiome change and enhanced fitness in *Daphnia magna*

Asa Motiei[1], Björn Brindefalk[2], Martin Ogonowski[1, 3], Rehab El-Shehawy[1], Paulina Pastuszek[2], Karin Ek[1], Birgitta Liewenborg[1], Klas Udekwu[2], Elena Gorokhova[1*]

*Corresponding Author: Elena.Gorokhova@aces.su.se

S1 Fig. Kaplan-Meier curves and estimates of survival data. Survival of *Daphnia magna* (proportion of survived individuals) exposed to Ciprofloxacin (0.01, 0.1 and 1 mg L^{-1}) and in the control during the 21-d exposure.

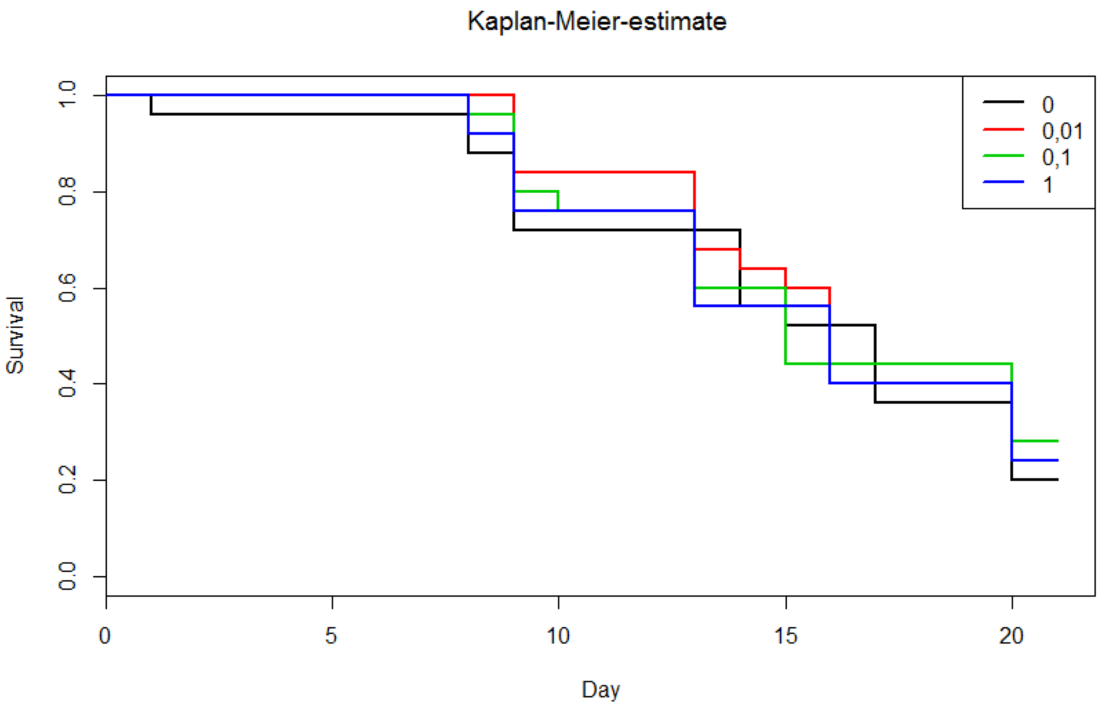

Supplement: S1 Fig — Survival of Daphnia magna exposed to Ciprofloxacin (0.01, 0.1 and 1 mg L-1) and in the control during the 21-d exposure. (PDF) [file pone.0214833.s010.pdf]
