# Supplementary material for: Disparate effects of antibiotic-induced microbiome change and enhanced fitness in Daphnia magna
Source: PLoS One. 2020 Jan 3;15(1):e0214833. doi: 10.1371/journal.pone.0214833 (PMC6941804; doi:10.1371/journal.pone.0214833)

S2 Fig. Neonate production in the exposed and non-exposed animals. Reproduction of *Daphnia magna* (brood size and time of reproduction) during a 21-d exposure to Ciprofloxacin (0.01, 0.1, and 1 mg L^{-1}) and the control. Note that the last clutch was estimated using both the offspring released and the embryos in the brood chamber at the termination of the experiment.

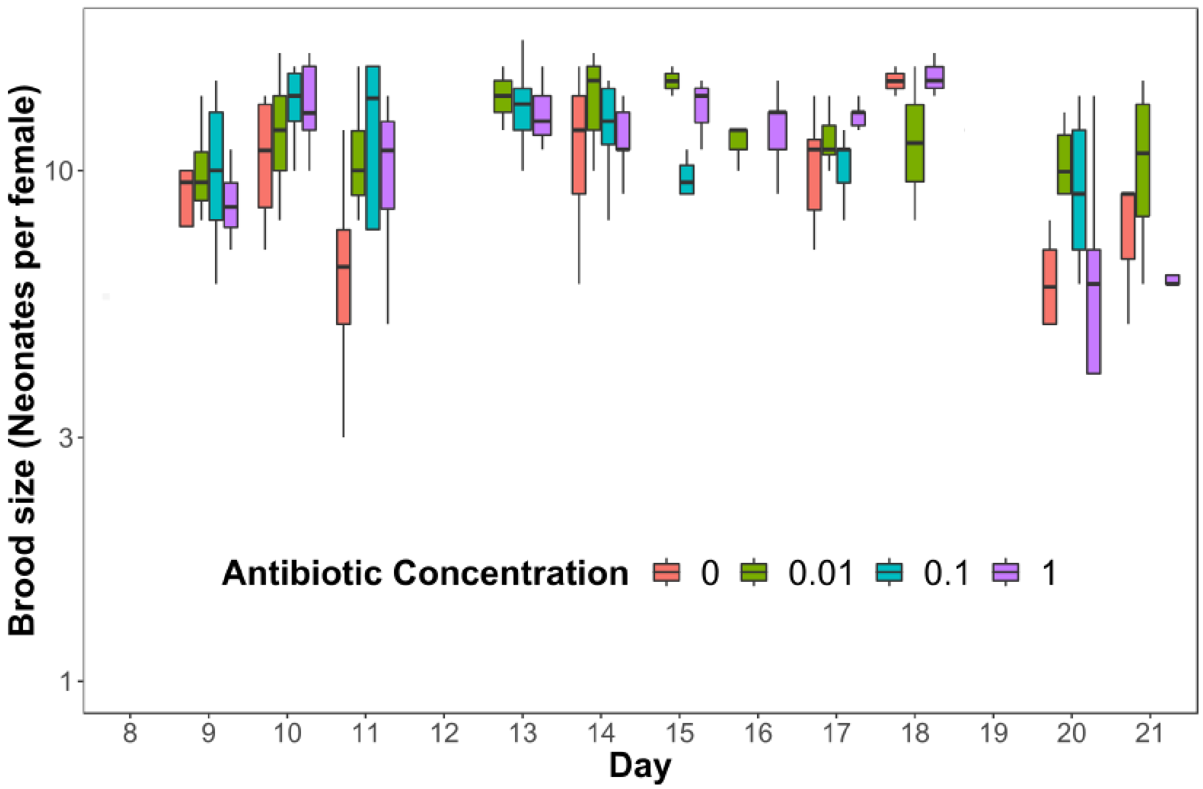

Supplement: S2 Fig — Reproduction of Daphnia magna (brood size and time of reproduction) during a 21-d exposure to Ciprofloxacin (0.01, 0.1, and 1 mg L-1) and the control. Note that the last clutch was estimated using both the offspring released and the embryos in the brood chamber at the termination of the experiment. (PDF) [file pone.0214833.s011.pdf]
