# Supplementary material for: Disparate effects of antibiotic-induced microbiome change and enhanced fitness in Daphnia magna
Source: PLoS One. 2020 Jan 3;15(1):e0214833. doi: 10.1371/journal.pone.0214833 (PMC6941804; doi:10.1371/journal.pone.0214833)

S3 Fig. Rarefaction curves for sequence data on the gut bacteria in *Daphnia magna*. Rarefaction curves show the cumulative number of unique OTUs as a function of sample size (number of reads for the 16S rRNA gene) for all individuals sampled in different treatments (Ciprofloxacin concentration, mg L^{-1}) and control during the experiment. Colors denote the clutch number.

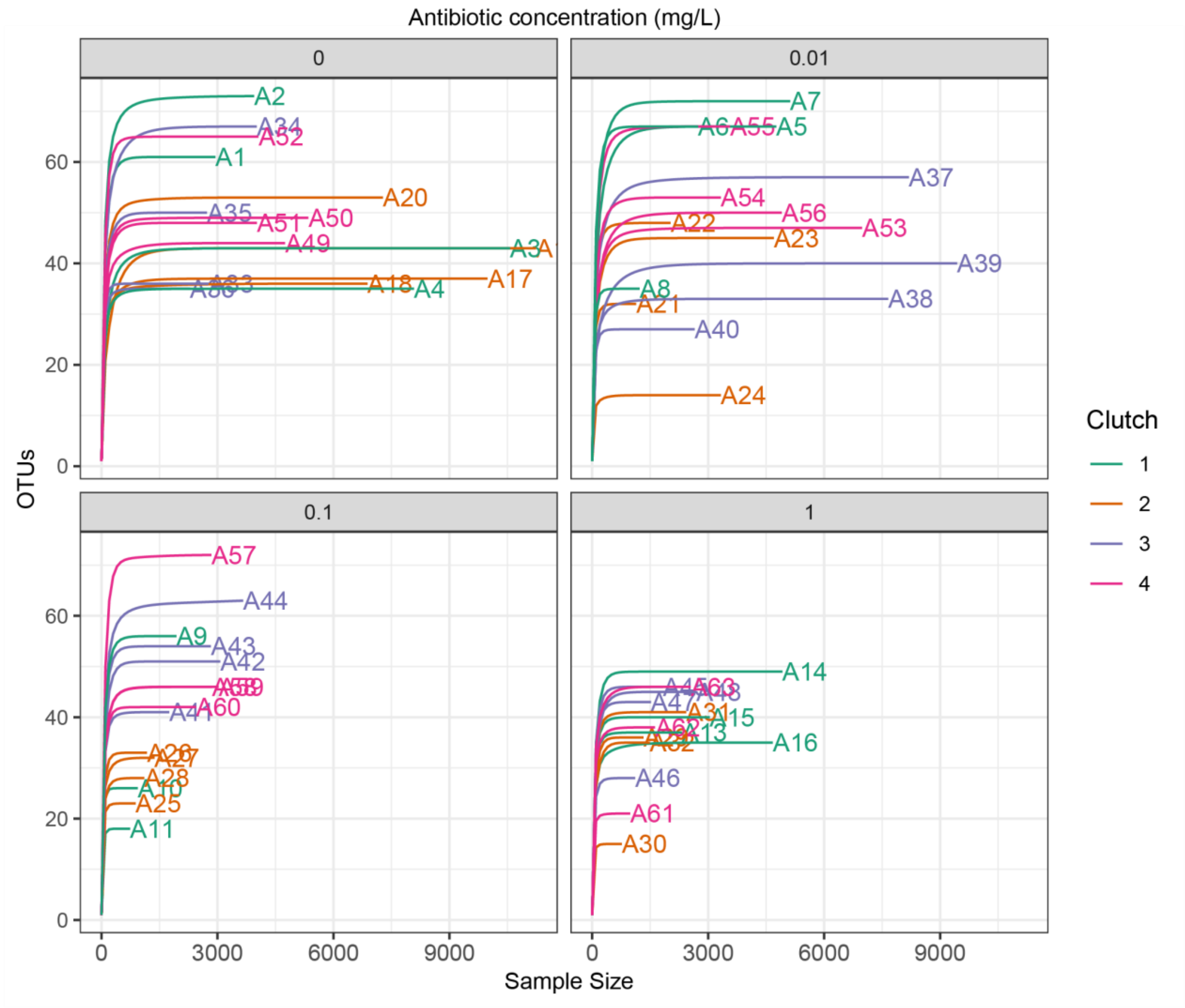

Supplement: S3 Fig — Rarefaction curves show the cumulative number of unique OTUs as a function of sample size (number of reads for the 16s rRNA gene) for all individuals sampled in different treatments (Ciprofloxacin concentration, mgL-1) and control during the experiment. Colors denote the clutch number. (PDF) [file pone.0214833.s012.pdf]
