# Supplementary material for: Disparate effects of antibiotic-induced microbiome change and enhanced fitness in Daphnia magna
Source: PLoS One. 2020 Jan 3;15(1):e0214833. doi: 10.1371/journal.pone.0214833 (PMC6941804; doi:10.1371/journal.pone.0214833)

S4 Fig. Relative abundance of bacterial taxa in the microbiome of *Daphnia magna* from the controls. The abundances are shown for the different taxonomy ranks: (a) Phylum, (b) Class, (c) Order, (d) Family, and (e) genus. Along the vertical axis, the data are grouped by clutch, 1 to 4, produced during the experiment.

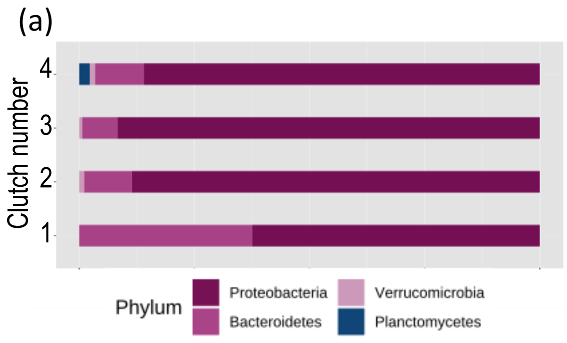

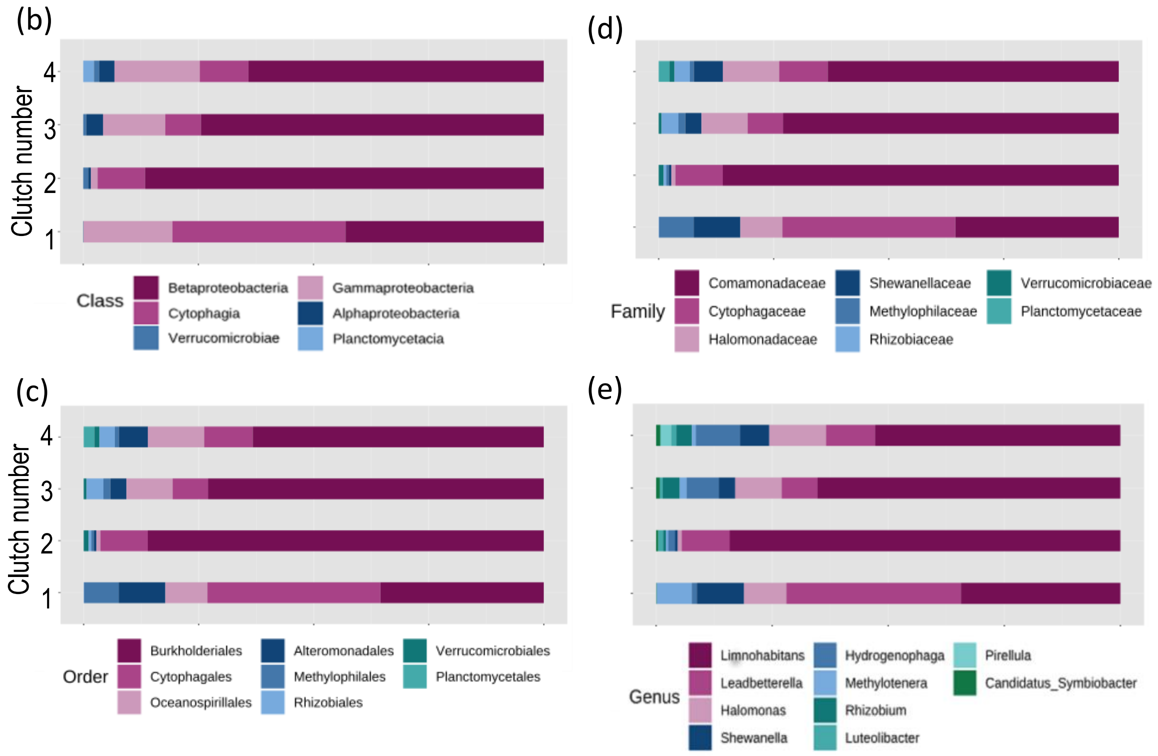

Supplement: S4 Fig — The abundances are shown for the different taxonomy ranks: (a) Phylum, (b) Class, (c) Order, (d) Family, and (e) Genus. Along the vertical axis, the data are grouped by the clutch, 1 to 4, produced during the experiment. (PDF) [file pone.0214833.s013.pdf]
