# Supplementary material for: Disparate effects of antibiotic-induced microbiome change and enhanced fitness in Daphnia magna
Source: PLoS One. 2020 Jan 3;15(1):e0214833. doi: 10.1371/journal.pone.0214833 (PMC6941804; doi:10.1371/journal.pone.0214833)

S5 Fig. The heatmap of the core microbiome in *Daphnia magna*. The dominant microbiome taxa across different taxonomic categories: (a) Phylum (b) Class, (c) Order, (d) Family, and (e) Genera, in *Daphnia magna* collected during the experiment.

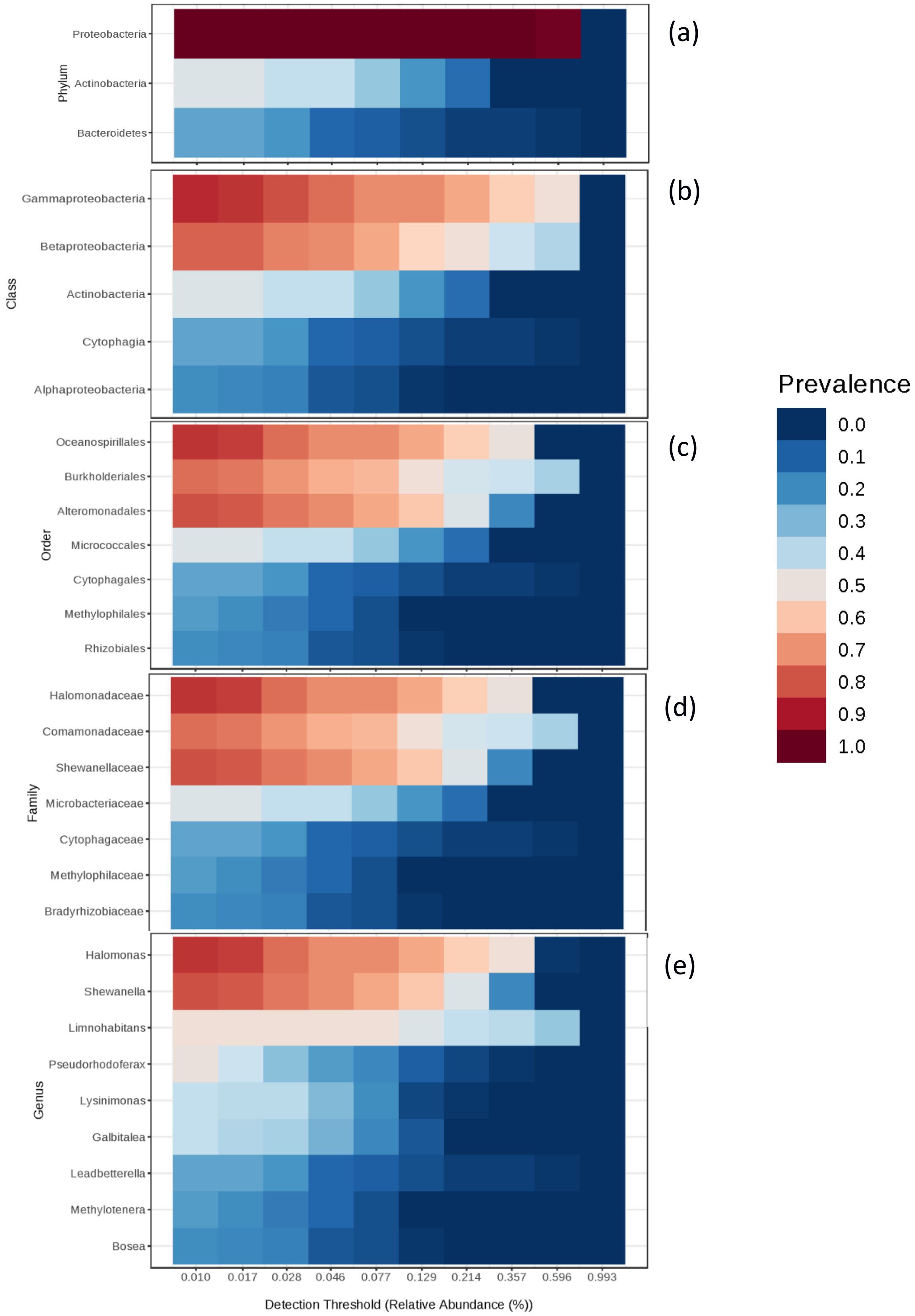

Supplement: S5 Fig — The heatmap of the core microbiome in Daphnia magna collected during the experiment across different taxonomic categories: (a) Phylum (b) class, (c) order, (d) family, and (e) genera. (PDF) [file pone.0214833.s014.pdf]
