# Supplementary material for: Disparate effects of antibiotic-induced microbiome change and enhanced fitness in Daphnia magna
Source: PLoS One. 2020 Jan 3;15(1):e0214833. doi: 10.1371/journal.pone.0214833 (PMC6941804; doi:10.1371/journal.pone.0214833)

S6 Fig. Variation in ORAC levels measured in *Daphnia magna* from different treatments. The total antioxidant capacity (ORAC, g Trolox eq./ g protein) was assayed in individual daphnids during the course of the experiment. The data are shown for the control (Concentration 0 mg L^{-1}) and each treatment (Ciprofloxacin concentration: 0.01, 0.1 and 1 mg L^{-1}). The regression line and the 95%-confidence interval (shaded area) are shown to indicate the overall direction of change over time in different treatments.

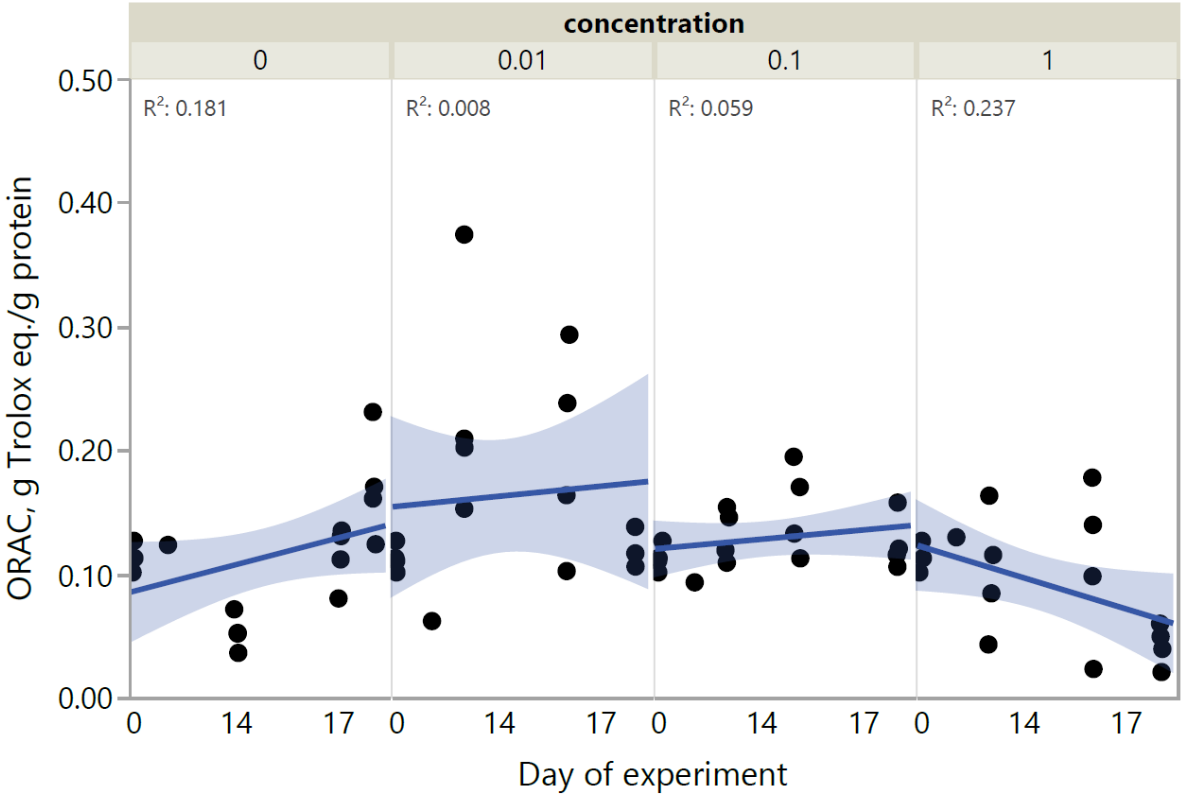

Supplement: S6 Fig — The total antioxidant capacity (ORAC, g Trolox eq./ g protein) was assayed in individual daphnids during the course of the experiment. The data are shown for the control (Concentration 0 mg L-1) and each treatment (Ciprofloxacin concentration: 0.01, 0.1 and 1 mg L-1). The regression line and the 95%-confidence interval are shown to indicate the overall direction of change over time in different treatments. (PDF) [file pone.0214833.s015.pdf]
