# Supplementary material for: Disparate effects of antibiotic-induced microbiome change and enhanced fitness in Daphnia magna
Source: PLoS One. 2020 Jan 3;15(1):e0214833. doi: 10.1371/journal.pone.0214833 (PMC6941804; doi:10.1371/journal.pone.0214833)

S7 Fig. Changes in relative abundance of Gram-positive (G+) and Gram-negative (G-) bacteria in response to Ciprofloxacin exposure. Fold-change of G- and G+ bacteria in gut microbiota of *D. magna* exposed to Ciprofloxacin (0 to 1 mg/L). For G+ bacteria at the order level, mostly increase in response to Ciprofloxacin was observed as shown for, for example, Actinobacteria (a) and Firmicutes (b). For G- bacteria, the responses were more divergent. For example, responses of *Pseudorhodoferax* (c) and *Hydrogenophaga* (d) families belonging to the same order Burkholderiales were the opposite.

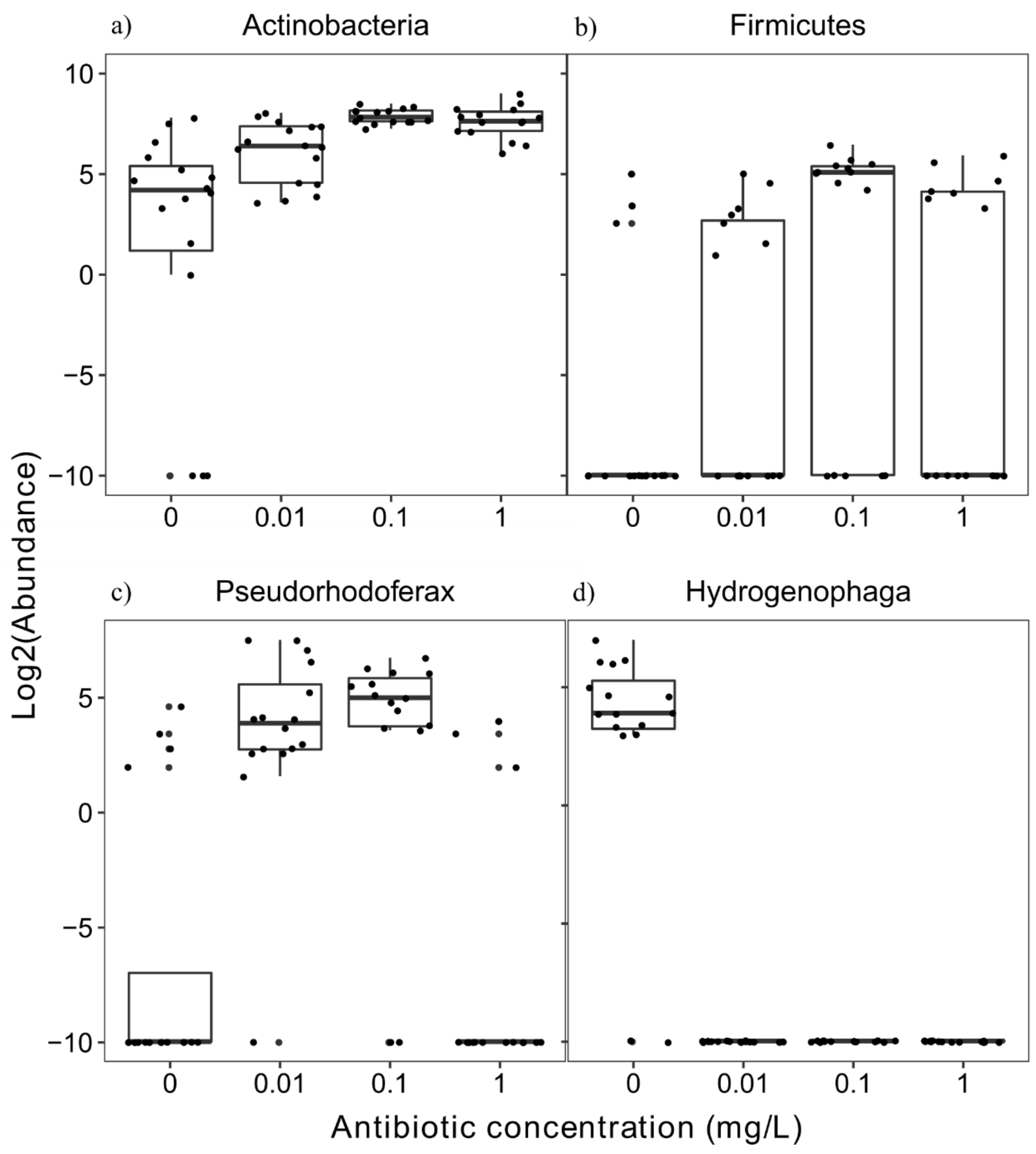

Supplement: S7 Fig — Fold-change of G- and G+ bacteria in gut microbiota of D. magna exposed to Ciprofloxacin (0 to 1 mg/L). For G+ bacteria at the order level, mostly increase in response to Ciprofloxacin was observed as shown for, for example, Actinobacteria (a) and Firmicutes (b). For G- bacteria, the responses were more divergent. For example, responses of Pseudorhodoferax (c) and Hydrogenophaga (d) families belonging to the same order Burkholderiales were the opposite. (PDF) [file pone.0214833.s016.pdf]
